# Diffusion MRI of the facial-vestibulocochlear nerve complex: a prospective clinical validation study

**DOI:** 10.1007/s00330-023-09736-4

**Published:** 2023-06-17

**Authors:** Jonathan Shapey, Sjoerd B. Vos, Laura Mancini, Brett Sanders, John S. Thornton, Jacques-Donald Tournier, Shakeel R. Saeed, Neil Kitchen, Sherif Khalil, Patrick Grover, Robert Bradford, Reuben Dorent, Rachel Sparks, Tom Vercauteren, Tarek Yousry, Sotirios Bisdas, Sebastien Ourselin

**Affiliations:** 1 School of Biomedical Engineering & Imaging Sciences, King’s College London, London, UK; 2 Department of Neurosurgery, King’s College Hospital, London, UK; 3 Wellcome/EPSRC Centre for Interventional and Surgical Sciences, University College London, London, UK; 4 Centre for Medical Image Computing, University College London, London, UK; 5 Neuroradiological Academic Unit, UCL Institute of Neurology, London, UK; 6 Lysholm Department of Neuroradiology, National Hospital for Neurology and Neurosurgery, Queen Square, London, UK; 7 Department of Neurophysiology, National Hospital for Neurology and Neurosurgery, Queen Square, London, UK; 8 Department of Neurosurgery, National Hospital for Neurology and Neurosurgery, Queen Square, London, UK; 9 The Ear Institute, University College London, London, UK; 10 The Royal National Throat, Nose and Ear Hospital, London, UK

**Keywords:** Magnetic resonance imaging, Diffusion magnetic resonance imaging, Cranial nerves, Vestibular schwannoma

## Abstract

**Objectives:**

Surgical planning of vestibular schwannoma surgery would benefit greatly from a robust method of delineating the facial-vestibulocochlear nerve complex with respect to the tumour. This study aimed to optimise a multi-shell readout-segmented diffusion-weighted imaging (rs-DWI) protocol and develop a novel post-processing pipeline to delineate the facial-vestibulocochlear complex within the skull base region, evaluating its accuracy intraoperatively using neuronavigation and tracked electrophysiological recordings.

**Methods:**

In a prospective study of five healthy volunteers and five patients who underwent vestibular schwannoma surgery, rs-DWI was performed and colour tissue maps (CTM) and probabilistic tractography of the cranial nerves were generated. In patients, the average symmetric surface distance (ASSD) and 95% Hausdorff distance (HD-95) were calculated with reference to the neuroradiologist-approved facial nerve segmentation. The accuracy of patient results was assessed intraoperatively using neuronavigation and tracked electrophysiological recordings.

**Results:**

Using CTM alone, the facial-vestibulocochlear complex of healthy volunteer subjects was visualised on 9/10 sides. CTM were generated in all 5 patients with vestibular schwannoma enabling the facial nerve to be accurately identified preoperatively. The mean ASSD between the annotators’ two segmentations was 1.11 mm (SD 0.40) and the mean HD-95 was 4.62 mm (SD 1.78). The median distance from the nerve segmentation to a positive stimulation point was 1.21 mm (IQR 0.81–3.27 mm) and 2.03 mm (IQR 0.99–3.84 mm) for the two annotators, respectively.

**Conclusions:**

rs-DWI may be used to acquire dMRI data of the cranial nerves within the posterior fossa.

**Clinical relevance statement:**

Readout-segmented diffusion-weighted imaging and colour tissue mapping provide 1–2 mm spatially accurate imaging of the facial-vestibulocochlear nerve complex, enabling accurate preoperative localisation of the facial nerve. This study evaluated the technique in 5 healthy volunteers and 5 patients with vestibular schwannoma.

**Key Points:**

• *Readout-segmented diffusion-weighted imaging (rs-DWI) with colour tissue mapping (CTM) visualised the facial-vestibulocochlear nerve complex on 9/10 sides in 5 healthy volunteer subjects.*

• *Using rs-DWI and CTM, the facial nerve was visualised in all 5 patients with vestibular schwannoma and within 1.21–2.03 mm of the nerve’s true intraoperative location.*

• *Reproducible results were obtained on different scanners.*

**Supplementary Information:**

The online version contains supplementary material available at 10.1007/s00330-023-09736-4.

## Introduction

Diffusion magnetic resonance imaging (dMRI) creates image contrast based on the relative diffusivity of water molecules in tissue and can enable visualisation of the brain’s neural tracts and connectivity [1]. Recently, there has been growing clinical interest in generating tractography of the cranial nerves to assist the clinical diagnosis of various neurological conditions and to inform the surgical planning of complex skull base neurosurgical procedures [2]. In particular, given the potentially disastrous complication of facial nerve palsy that too often occurs as a result of vestibular schwannoma (VS) surgery [3], there has been specific interest in delineating the facial nerve in the context of VS surgery in order to inform surgeons of its location preoperatively. In the presence of tumour, high-resolution T2-weighted (hrT2) imaging does not permit the visualisation of facial-vestibulocochlear complex (VII/VIII complex) and no diffusion method has yet been shown capable of reliably delineating the facial nerve from within the larger VII/VIII complex [2].

Currently, state-of-the-art acquisition and tractography methods are not sufficiently spatially accurate to delineate these small nerves in the VII/VIII complex. This is of particular concern when trying to precisely locate small structures such as the facial nerve intraoperatively. In nearly all previous patient studies involving posterior fossa tumours, diffusion-weighted imaging (DWI) with conventional single-shot echo planar imaging (EPI) was used to image the region of interest in an attempt to delineate the VII/VIII complex and other associated cranial nerves [2]. Readout-segmented DWI (rs-DWI) using multi-shot EPI may be used to improve the image quality and resolution of dMRI data [4, 5], and reduce partial volume effects. A limited number of studies have utilised this method to image the VII/VIII complex in healthy volunteers [6, 7] and to delineate temporal bone tumours such as cholesteatoma more clearly [8]. An alternative approach is available using multi-shell dMRI data from multiple *b*-value acquisitions to better characterise intra-voxel partial voluming, but no previous studies have examined using a combined multi-shell rs-DWI sequence to image the VII/VIII complex.

Aside from the acquisition options described above, there are also various ways of analysing the dMRI data once acquired [2]. Most previous studies that performed tractography of the cranial nerves used a deterministic approach selecting two regions of interest (ROIs) to seed and select the fibre tracts, typically the internal auditory meatus (IAM) brainstem surface [2]. More recently, others have sought to improve the accuracy of cranial nerve tractography by employing a “superselective” diffusion tensor method [9], using diffusion spectrum imaging (DSI) [10, 11] or constrained spherical deconvolution (CSD) [12, 13] and through the use of probabilistic tractography [7, 12].

Colour tissue maps (CTM) may be generated from DWI using a multi-tissue CSD method to perform an unsupervised estimation of white matter (WM), grey matter (GM), and cerebrospinal fluid (CSF) tissue fractions [14]. Three-tissue CTM have been used to examine white matter changes in dementia [15, 16], stroke [17], and the optic pathway in patients with multiple sclerosis optic neuritis [18]. No previous study has applied this image-processing method to visualise and examine structures as small as the facial and vestibular nerves.

In this study, we optimised a multi-shell rs-DWI protocol and developed a novel post-processing pipeline to generate CTM and cranial nerve probabilistic tractography of the VII/VIII complex within the internal auditory meatus and skull base region in healthy volunteers and in patients with a VS. We tested the reproducibility of the acquisition protocol and processing pipeline in healthy volunteers and then analysed the accuracy of the method in delineating the facial nerve in patients undergoing VS surgery using neuronavigation and tracked electrophysiological recordings.

## Materials and methods

This prospective study was approved by the institution’s local ethics committee (18/LO/0532 and 09/H0716/18) and registered in a public trials registry (NCT04128345).

### Subjects

Ten subjects were enrolled in the study including 5 healthy volunteers (V1–5) and 5 patients (P1–5) with a VS scheduled for surgery. Adult patients aged 18–85 years, with a unilateral VS scheduled for surgery either via a translabyrinthine or retrosigmoid approach who were willing and able to provide written informed consent, were eligible to participate in the study. Patients with a history of neurofibromatosis type II, previous treatment or surgery for a posterior fossa brain tumour, ipsilateral ear, or facial surgery, or any contraindication to MR imaging were excluded.

### Image acquisition

All subjects were imaged using a Siemens 3 T Prisma-fit scanner with a 64-channel head coil at the National Hospital for Neurology and Neurosurgery (centre 1: NHNN). To test reproducibility, all healthy subjects were scanned twice on different days and two subjects were also scanned on a second Siemens 3 T Prisma scanner at a separate institution (centre 2: Chenies Mews Imaging Centre, London). Patients were typically imaged 2–4 weeks before surgery.

The following sequences were acquired in all subjects: (1) T1-weighted (T1) MPRAGE anatomical scan of the whole brain with an acquired isotropic voxel size of 1 × 1 × 1 mm (TR (repetition time) = 2000 ms, TE (echo time) = 2.01 ms, TI (inversion time) = 880 ms, TA (acquisition time) = 4′53″); (2) high-resolution T2-weighted (hrT2) small field of view imaging inner volume excitation (Siemens ZOOMit sequence) with an acquired isotropic voxel size of 0.5 × 0.5 × 0.5 mm (TR = 1000 ms, TE 127 ms, TA = 3′35″) through the region of the internal auditory meatus; and (3) RESOLVE (REadout Segmentation Of Long Variable Echo-trains) rs-DWI sequence with the following parameters: 7 shots, an acquired isotropic voxel size of 1.2 × 1.2 × 1.2 mm, using three *b*-values (*b* = 0, *b* = 700, *b* = 2000 s/mm^2^), with 5, 17, and 30 directions per *b*-value, respectively (TR = 4300 ms, TE = 60 ms, TA = 31′34″). All patients also had a contrast-enhanced volumetric T1 MPRAGE scan with an acquired isotropic voxel size of 0.5 × 0.5 × 0.5 mm (TR = 2020 ms, TE = 1.65 ms, TI 1100 ms, TA = 5′19″).

### Image processing

Images were analysed using MRtrix3 software^1^ [19]. Pre-processing steps involved denoising the diffusion data and removing Gibbs-ringing artefacts using the MRtrix3 ‘dwidenoise’ [20] and ‘mrdegibbs’ [21] tools, respectively. A geometric mismatch between the structural images and the diffusion image was estimated with the FSL ‘topup’ tool [22] and all diffusion sequences were corrected for eddy-current and motion distortions using the FSL ‘eddy’ tool [23].

For subsequent post-processing image analysis, a brain mask was formed using the MRtrix3 ‘dwi2mask’ command, using ‘dwi2tensor’ and ‘tensor2metric’ to extract DTI parametric maps such as fractional anisotropy (FA) and mean diffusivity (MD) [19]. The diffusion signal for a single fibre was estimated using the ‘dwi2response’ command using an unsupervised multi-tissue estimation of WM, GM, and CSF response functions [24] and then incorporated into a multi-shell multi-tissue CSD [14] analysis from which fibre orientation distributions (FOD) were computed using the ‘dwi2fod’ command. CTM of the skull base region were generated based on the estimated tissue fractions of the three FODs. As originally demonstrated in Jeurissen et al [14], a default colour scheme (CSF: red, GM: green, WM: blue) is generated^2^; however, we altered the colours assigned to each tissue type to improve clarity in the region of interest. The final colour maps were colour coded as follows: WM: red, GM: green, CSF: blue.

### Probabilistic tractography

In line with previous work, seeds were placed within the IAM and along the brainstem border [2]. Regions of interest (ROIs) were drawn on the hrT2 sequence and then registered to the diffusion data using NiftyReg’s affine registration ‘reg_aladin’ tool (https://github.com/KCL-BMEIS/niftyreg/wiki) [25]. The IAM ROI were typically drawn on a single sagittal slice, medial to the cochlea where all four branches of the VII/VIII complex could be visualised individually, and the brainstem border was drawn on sequential axial slices. Probabilistic tractography was performed using the ‘tckgen’ command with the iFOD2 algorithm in MRtrix3 [26]. 1000 streamline tracks were selected and experiments were aborted if no tracts were detected from 100,000 streamlines.

All computations were performed on a MacBook Pro (2017) with a 3.1 GHz Intel Core i5 processor.

### Healthy volunteer analysis

Two neuroradiologists inspected the processed imaging data and independently rated the 5 healthy volunteer datasets (V1-5) scoring the following criteria: (1) visualisation of the VII/VIII complex within the CSF cistern and IAM on (a) hrT2 and (b) CTM; and (2) correlation of the CTM and tractography results to the subject’s anatomical (hrT2) imaging. Visibility of the nerves was scored using a 5-point scale: (1) not at all visible, (2) visible on < 25% of its course, (3) visible on 25–50% of its course, (4) visible on 50–75% of its course, (5) visible on 75–100% of its course. Correlation of the CTM and tractography results to the subject’s anatomical (hrT2) imaging was also scored using a 5-point scale: (1) completely different location and orientation to anatomical imaging, (2) broadly similar location and orientation but poorly aligned with anatomical imaging, (3) correct location and orientation but mismatch in parts, (4) correct location and orientation and very closely aligned, (5) completely aligned with anatomical imaging.

### Patient analysis

To replicate the current clinical workflow, an experienced Clinical Scientist and Clinical Research Fellow (L.M. and J.S.) were trained to interpret the CTM and segment the facial nerve in patients with VS. Training was provided using cases not included in the final data analysis. Segmentations were performed manually using freely available open-source software ITK Snap (http://www.itksnap.org/pmwiki/pmwiki.php) [27]. Only portions of the nerve that could be confidently segmented were labelled as nerve. The assessors used a 5-point scale to score: (1) ease of segmenting the nerve, and (2) confidence that the segmentation was correct.

Visibility of the facial nerve was scored using a 5-point scale by two independent neuroradiologists (T.Y. and S.B.) using the same method as above. Each neuroradiologist then correlated the subject’s CTM, tractography, and both nerve segmentations to the subject’s anatomical (ceT1 and hrT2) imaging, using a 5-point scale. Finally, a consensus segmentation was reached among the neuroradiologists.

The surgical treatment of patients enrolled in this study was not altered and the operating surgeon was blinded to the dMRI results. Prior to surgery, the patient’s contrast-enhanced volumetric T1 MPRAGE scan was uploaded onto a Medtronic Stealth neuronavigation system. The dMRI results were not uploaded onto the navigation system. In line with standard practice, continuous intraoperative recording of spontaneous electromyographic activity and observed responses to electrical stimulation of the facial nerve were performed throughout the procedure. The neurostimulation probe was fitted with a SureTrak™ instrument marker to measure the location of all stimulation points with the stimulation amplitude (mA) and distal output also recorded. In addition to the tracked electrophysiological recordings, the operating surgeon recorded the intraoperative location of the facial nerve, if seen under direct vision. This information was collected intraoperatively through direct measurement and observation constituted the study’s ground truth (Table 1).

**Table 1:** Interpretation of colour tissue mapping and facial nerve segmentation in patients with a VS: a qualitative assessment of task difficulty between two assessors and a quantitative assessment of segmentations. CTM interpretation 5-point score: (1) very difficult, (2) difficult, (3) neutral, (4) easy, (5) very easy. Confidence 5-point score: (1) not at all confident, (2) a little unsure, (3) neutral, (4) confident, (5) very confident. *CTM*, colour tissue mapping; *Ann*., annotator; *seg.*, segmentation; *ASSD*, average symmetric surface distance; *HD-95*, 95% Hausdorff distance

Subject	CTM interpretation		Confidence of segmentation		Quantitative assessment	
	Ann. 1	Ann. 2	Ann. 1	Ann. 2	ASSD (mm)	HD-95 (mm)
P1	4	2	5	1	1.28	5.41
P2	3	1	4	1	0.99	5.96
P3	4	1	4	1	0.60	1.96
P4	3	1	4	1	1.67	6.12
P5	3	2	5	2	1.01	3.64

For quantitative analysis of the segmentations, we used two surface metrics—the average symmetric surface distance (ASSD) and Hausdorff distance (HD)—to measure the spatial discrepancy (in millimetres) between boundaries of each segmentation compared to the consensus results [28]. The ASSD is determined by the average spatial distance between the border voxels of the automated segmentation results and the ground truth. A lower ASSD value indicates a better agreement, with ASSD = 0 representing a perfect agreement in the segmentation boundary (Suppl Figure 1) [28]. The maximum HD is defined as the maximal distance from a point on a boundary to the nearest point on the other boundary. For the 95% HD (HD-95), 95% of the voxels of a segmentation boundary are within the HD-95 distance of the other boundary (Suppl Figure 2) [28].

## Results

### Healthy volunteer results

**Fig. 1:**
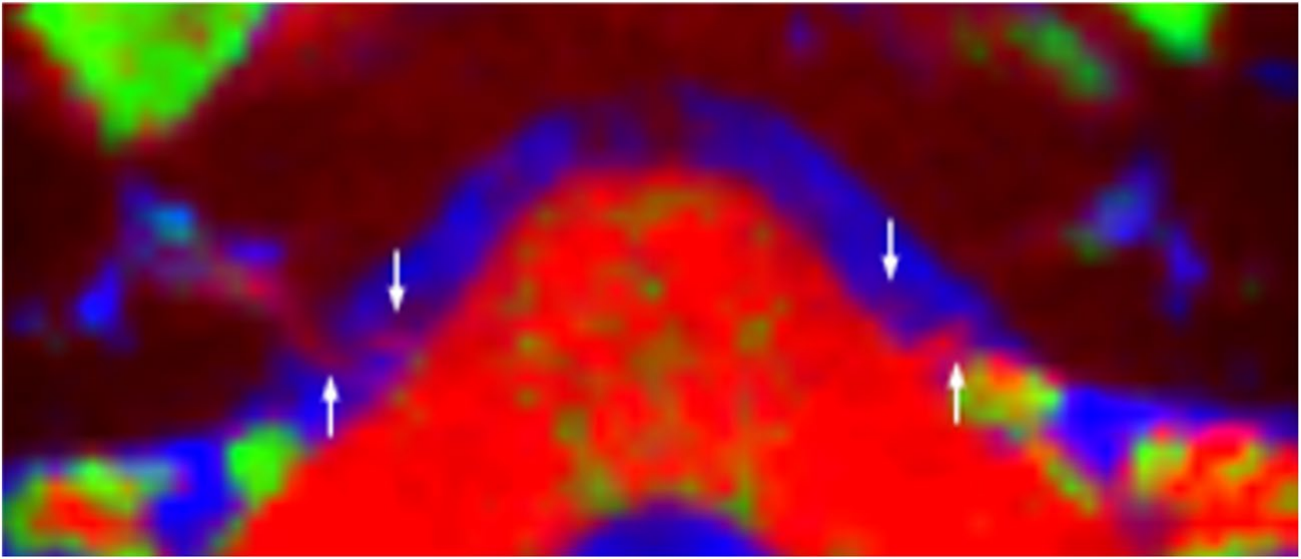
Colour tissue mapping of health volunteer subject (V1). The facial and vestibular nerves (arrows) are visible on both sides

The VII/VIII complex was visualised within the CSF cistern and IAM on both sides in all healthy volunteer subjects on hrT2 imaging. Individual nerves were visible within every subject’s IAM and the facial nerve was anatomically distinct from the vestibular nerve within the CSF cistern and IAM. Using CTM alone, the VII/VIII complex was visualised in 9 out of 10 sides (Table 2). To improve the specificity of imaging individual cranial nerves, a multi-shell rs-DWI acquisition was used to overcome the limited spatial resolution of single-shell acquisitions (Suppl Figure 3). Using this technique, individual cranial nerves were visualised in 3 of the 10 sides (for example subject V1, Fig. 1). Comparable results were obtained irrespective of the scanner used to obtain the imaging.

**Table 2:** dMRI of the facial-vestibulocochlear nerve complex in healthy volunteer subjects. *hrT2*, high-resolution T2-weighted imaging; *CTM*, colour tissue mapping. No difference in results between scan centres with the exception of case V1-right side (*). In this case, < 25% of the subject’s right-sided cranial nerves (both CN VII and VIII) could be visualised on the images obtained at centre 2, compared to 25–50% of CN VII and 50–75% of CN VIII that were visualised on images obtained at centre 1. *WCO*, whole complex only

Subject	Side	Visualisation on hrT2			Visualisation on CTM		
		VII/VIII	VII	VIII	VII/VIII	VII	VIII
V1	L	✓	✓	✓	×	×	×
	R	✓	✓	✓	✓	25–50%*	50–75%*
V2	L	✓	✓	✓	✓	WCO	WCO
	R	✓	✓	✓	✓	WCO	WCO
V3	L	✓	✓	✓	✓	WCO	WCO
	R	✓	✓	✓	✓	WCO	WCO
V4	L	✓	✓	✓	✓	WCO	WCO
	R	✓	✓	✓	✓	WCO	WCO
V5	L	✓	✓	✓	✓	25–50%	50–75%
	R	✓	✓	✓	✓	25–50%	50–75%

**Fig. 2:**
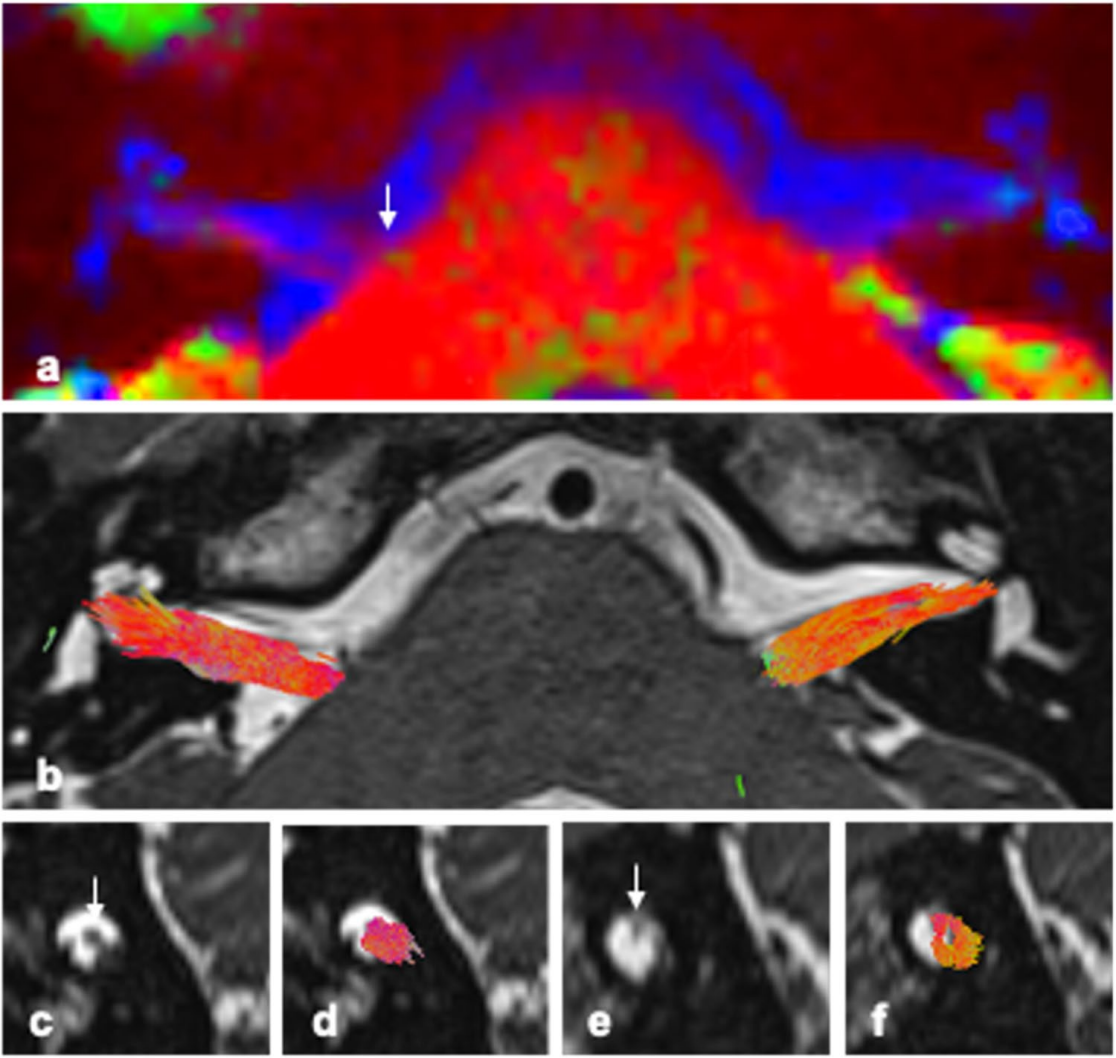
Colour tissue mapping and probabilistic tractography of CN VII/VIII complex in a healthy subject (V1). **a** Representative axial CTM image at level of IAM; **b** representative axial hrT2 with tractography at level of IAM; **c** to **f** sagittal views of the porus/IAM: **c** hrT2 image of right IAM/porus, **d** hrT2 of right IAM/porus with tractography overlaid, **e** hrT2 image of left IAM/porus, **f** hrT2 of left IAM/porus with tractography overlaid. *White arrow*: facial nerve highlighted on CTM or anatomical image (when visible on representative image)

CTM and tractography results aligned well with the subject’s hrT2 anatomical imaging (Fig. 2). Eighty-three percent of CTM results (20/24 sides) and 88% of tractography results (21/24 sides) were graded as being in the correct location with the correct orientation (score 3 +). The facial nerve could be distinguished separately from within the CN VII/VIII complex in 79% of cases within the IAM but was less clear within the cisternal segment.

### Patient results

**Fig. 3:**
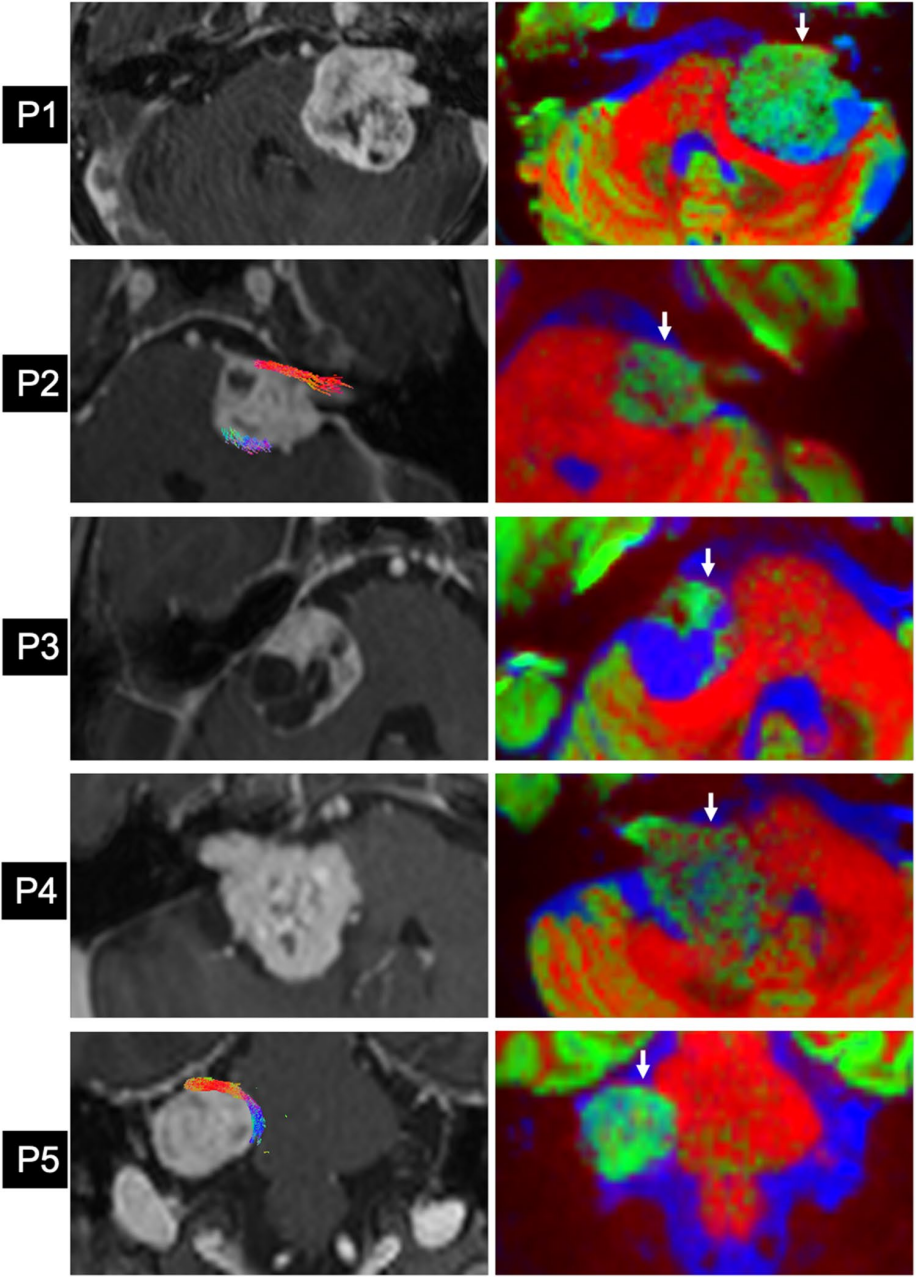
Colour tissue mapping and tractography of the facial nerve in patients with vestibular schwannoma (P1–P5). *Left*: contrast-enhance T1-weighted (ceT1) MRI scan of vestibular schwannoma. Tractography results overlaid on P2 and P5 (tractography not successful in other patients). P1–4: axial ceT1 MRI, P5: coronal ce-T1 MRI. *Right*: corresponding CTM image delineating the facial nerve (arrow). Colour legend: *red*, WM; *green*, GM/tumour; *blue*, CSF/cystic fluid

All patients were successfully imaged using the study’s MRI protocol and CTM was generated in all patients but probabilistic tractography was only successful in 2 patients (P2 and P5) (Fig. 3). There was variability between assessors in their assessment of how difficult a task it was to interpret the CTM and perform segmentation (Table 1). The mean ASSD between the annotators’ two segmentations was 1.11 mm (SD 0.40) and the mean Hausdorff 95% distance was 4.62 mm (SD 1.78) (Table 1, Fig. 3).

Two consultant neuroradiologists working in consensus found that the availability of CTM improved their ability to determine the location of the facial nerve in patients with a VS in 4 out of 5 cases (Table 3, Suppl Figure 4). In the one case (P5) where there was no improvement in visibility using CTM, it was already possible to determine the location of the facial nerve using hrT2 imaging. Using a 5-point score to describe the extent of nerve visibility, there was an average 2-point improvement in facial nerve visibility scores when using CTM, and over 50% of the course of the facial nerve could be visualised in 4 out of 5 patients. The remaining case (P3), in which the nerve was difficult to visualise even with CTM, was in a patient with a cystic VS.

**Table 3:** Visualisation of CN VII in patients with VS using CTM and hrT2: an independent. Assessment. Visibility 5-point scale: (1) not at all visible, (2) visible on < 25% of its course, (3) visible on 25–50% of its course, (4) visible on 50–75% of its course, (5) visible on 75–100% of its course. *hrT2*, high-resolution T2-weighted imaging; *CTM*, colour tissue mapping

Subject	Side	hrT2 only	CTM only	hrT2 & CTM
P1	L	2	4	4
P2	L	1	5	5
P3	R	2	3	3
P4	R	2	4	4
P5	R	5	4	5

**Fig. 4:**
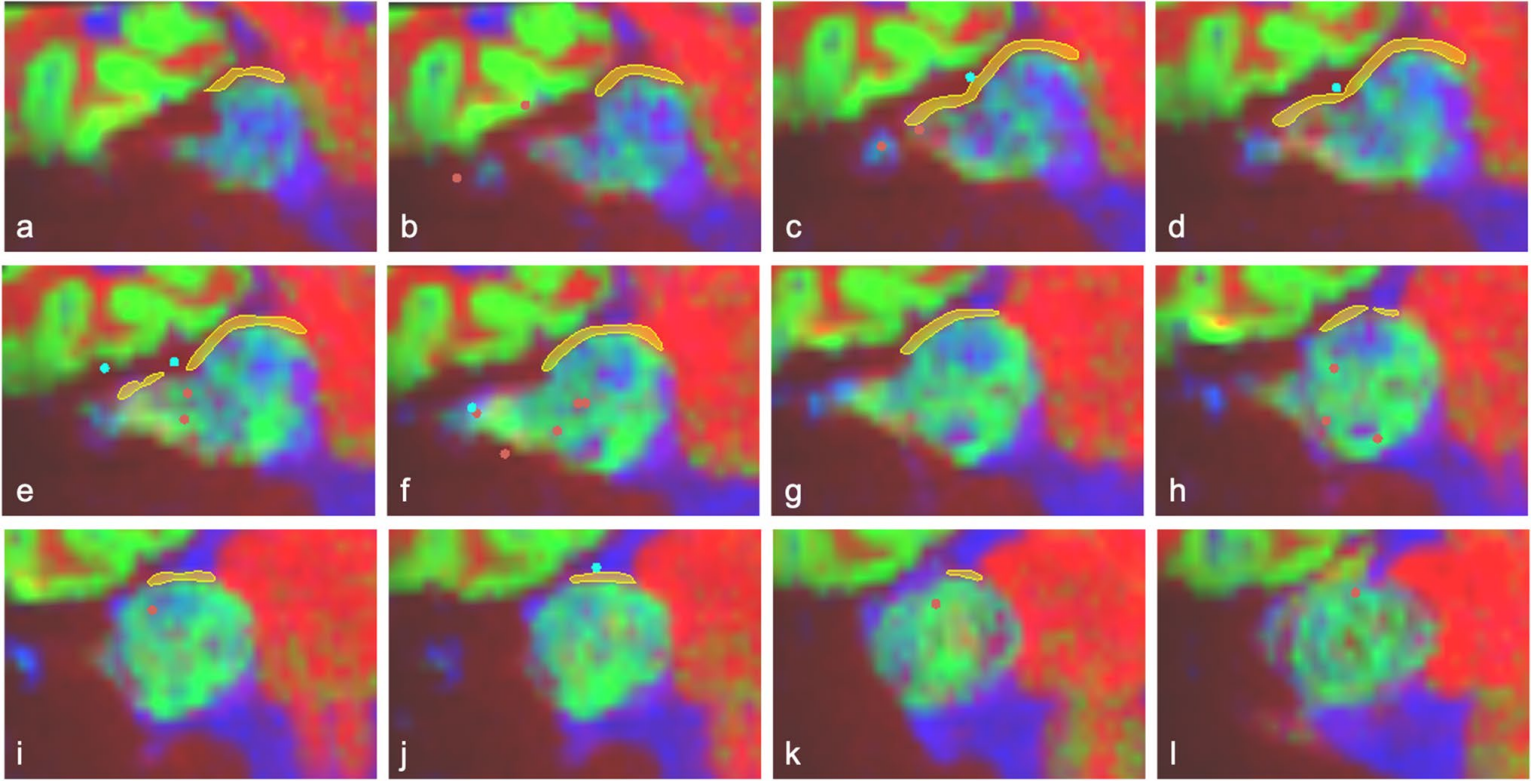
Navigated neurostimulation points with respect to facial nerve as manually segmented on colour tissue mapping (CTM). Patient P5 with right-sided vestibular schwannoma with facial nerve coursing anterosuperiorly over the tumour. Sequential coronal CTM slices through segmented nerve (**a**) anterior through to (**l**) posterior. *Yellow*: segmented facial nerve; *blue:* positive neurostimulation points at 0.11 mA; *red*: negative neurostimulation points

**Fig. 5:**
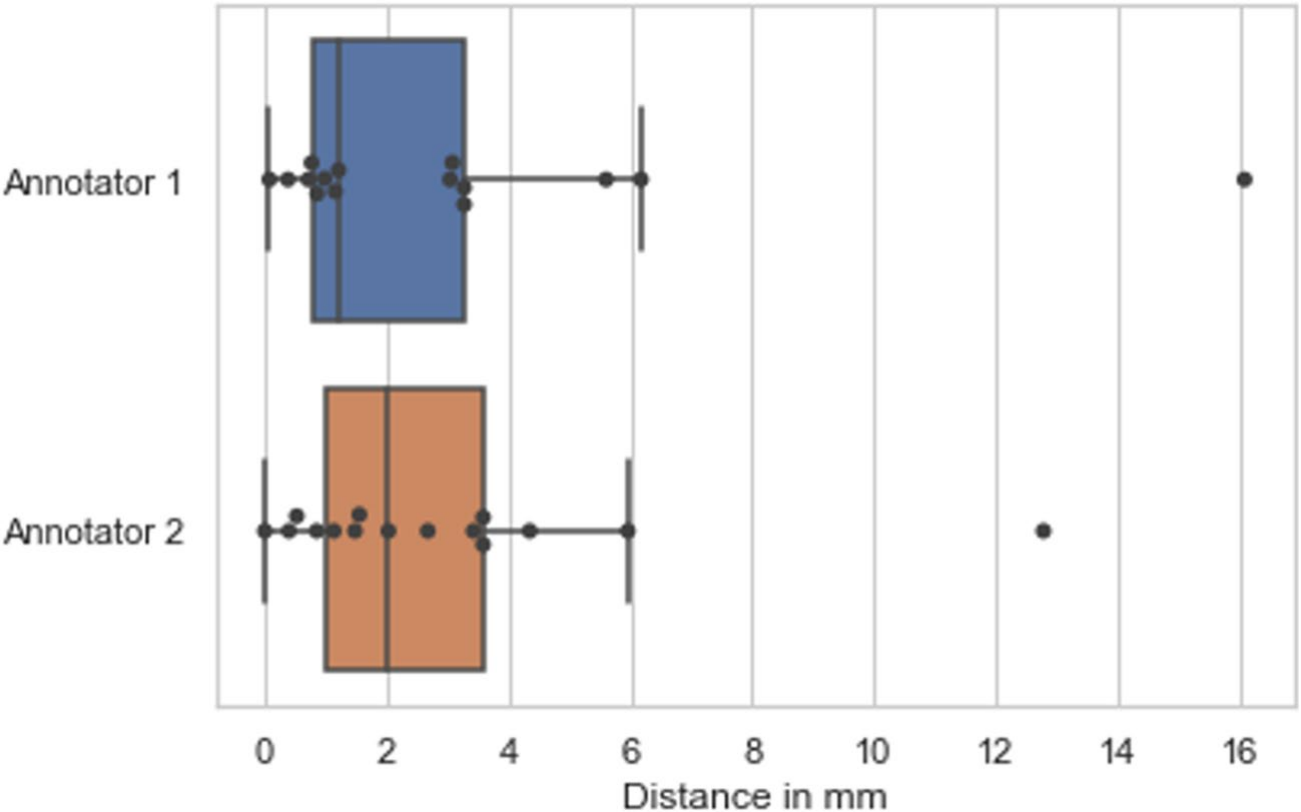
Distance of facial nerve segmentation from positive neurostimulation points. Box plot indicating median with IQR

Navigated intraoperative neuromonitoring and neurostimulation were performed in 4 cases: P1, P2, P3 and P5 (Table 4, Fig. 4, Fig. 5). Due to clinical urgency, P4’s surgery had to be expedited with standard non-navigated intraoperative neuromonitoring of the facial nerve used instead of the research protocol. In every case, a portion of the facial nerve was visualised intraoperatively by the operating surgeon and this segment was found to be in the same location as depicted preoperatively using dMRI. In all 4 patients for whom navigated neurostimulation data was obtained, not one *negative* neurostimulation point was located along the final consensus segmentation of the facial nerve. The median distance from the nerve segmentation to a positive stimulation point was 1.21 mm (IQR 0.81–3.27 mm) and 2.03 mm (IQR 0.99–3.84 mm) for annotators 1 and 2 respectively (Fig. 3). Probabilistic tractography was only obtained in two patients using a FOD-threshold of 0.05 (Table 4).

**Table 4:** Intraoperative validation of dMRI facial nerve results. *Surg. Obs.*, location of the facial nerve as observed by the operating surgeon during surgery; *Ant*., anterior; *Mid*., middle; *Sup*., superior; *Inf*., inferior; *S*_T_, total number of navigated neurostimulation points; *S*_N_, number of negative navigated neurostimulation points; *S*_P_, number of positive navigated neurostimulation points; *Tract*., generation of probabilistic tractography; *FOD-t*, fibre orientation density (FOD) threshold used to generate probabilistic tractography

Subject	Side	Surg. Obs	Stimulation points					Dist. FN seg	Tract
			*S*_T_	*S*_N_	(mA)	*S*_P_	(mA)	(mm)	(FOD-t)
P1	L	Ant. Mid	29	29	0.5	0	N/A	N/A	×
P2	L	Ant. Inf	15	1	0.02	1	0.02	16.08	✓ (0.05)
				4	0.50	1	0.12	1.15	
				7	0.52				
				1	1.00				
P3	R	Ant. Inf	17	1	0.02	1	0.02	4.35	×
				4	0.50	1	0.12	5.96	
				9	0.52				
				1	1.00				
P4	R	Ant. Mid	–	–	–	–	–	–	×
P5	R	Ant. Sup	47	32	0.11	15	0.11	0 to 3.58	✓ (0.05)

## Discussion

In the presence of a tumour, hrT2 imaging does not permit the visualisation of the adjacent facial nerve and current dMRI and tractography methods capable of delineating the facial nerve are unreliable [2]. Previous studies using dMRI have not shown specificity for the facial nerve, instead providing a reconstruction of the VII/VIII complex [2]. Consequently, it remains difficult to delineate the facial nerve in the presence of posterior fossa tumours such as VS. Furthermore, no study has objectively assessed the accuracy of dMRI or tractography results in these cases. In this work, we assessed the accuracy and reproducibility of a novel rs-DWI sequence and processing pipeline for imaging the VII/VIII complex in both healthy volunteers and patients with VS.

This study demonstrated that our novel rs-DWI acquisition and processing pipeline may be used to image the facial-vestibulocochlear complex in healthy volunteers and patients. We demonstrated that results could be reproduced on different MRI scanners. CTM and tractography results aligned well with hrT2 anatomical imaging (Table 2, Fig. 4), and using CTM alone, the VII/VIII complex could be visualised in 90%. However, visualising the individual nerves of the VII/VIII complex remains a complex task. Our multi-shell acquisition partly overcomes the limited spatial resolution inherent with single-shell acquisitions. The image voxel size used in the diffusion sequence was close to the diameter of the facial nerve meaning that partial volume effects could influence results. However, individual facial and vestibulocochlear nerves were visualised in 30% of cases. CTM and tractography of the VII/VIII complex aligned well with the subjects’ anatomical imaging but it was difficult to visualise the individual facial and vestibular nerves within the cisternal segment using CTM alone. This is probably due to the size of the respective nerves and the fact that they are surrounded by a higher volume of CSF within the cisternal segment compared to the IAM. As such, where possible, we would recommend that multi-modality imaging [29] including co-registered hrT2 anatomical imaging be used to localise individual cranial nerves within the CSF cistern prior to performing further analysis of the diffusion data relating to a specific nerve.

Patient results demonstrated that using CTM improves the robustness of visualising the facial nerve in the presence of a VS compared to using hrT2 and tractography alone. This has the potential to significantly improve the preoperative planning and subsequent safety of VS surgery. Probabilistic tractography was successfully generated in the two patients with a moderate-sized solid tumour; however, it struggled in very large or cystic tumours (P1, P3, and P4) (Fig. 2), probably because the nerve was extremely thin in parts causing isolated areas of signal drop out and thus a failure to generate a continuous streamline tract.

Despite the difficulties in generating facial nerve tracts, there was good agreement in CTM interpretation and segmentation between the two independent annotators although qualitative results demonstrated significant variation in the confidence of their interpretation of the results (Table 1). The contouring and segmentation of complex small brain structures is a complex task associated with a learning curve [30]. A longer period of training over more cases is therefore likely to improve an annotator’s confidence in interpreting the data.

Intraoperative validation of the dMRI results confirmed a high level of accuracy. For annotator 1, the nerve segmentation was within 1.21 mm from the actual position of the facial nerve and for annotator 2 the segmentation was within 2.03 mm of the nerve (Fig. 5). There was one outlier in the results, occurring at the same point for both annotators, corresponding to point 12 in P2 (Fig. 5). In this case, surgery was performed via a translabyrinthine approach and the facial nerve was identified laterally in the IAM. Given that this area lies within the temporal bone, it was not well visualised on dMRI and CTM and, thus, was not segmented.

This study has several limitations. This proof-of-concept study successfully demonstrated that rs-DWI may be used to acquire spatially accurate dMRI data of the cranial nerves within the posterior fossa; however, the small number of patients means it is underpowered to observe small effect sizes. The small inherent registration error of clinical neuronavigation systems may also represent a minor limitation in the method used to quantify the facial nerve location. Finally, all imaging data was processed and assessed by a small number of experts; further work is therefore needed to determine if the techniques and results reported here may be reproduced at other institutions.

This study demonstrated that CTM may be able to delineate the location of the facial nerve in patients with a VS and may be particularly advantageous when tumour morphology prohibits probabilistic tractography from being accurately generated. Further work involving a larger prospective clinical study is needed to evaluate the effectiveness of using CTM and tractography results of the facial nerve intraoperatively to guide surgical resection.

### Supplementary Information

Below is the link to the electronic supplementary material.

Supplementary file1 (PDF 1356 KB) **Suppl Figure 1**: Average Symmetric Surface Distance (ASSD) and Mean Average Surface Distance (MASD). The Euclidean distance between boundary pixels $a$ and $b$ is defined as $d(a,b)$. Only True Positive (TP) are considered. Figure courtesy of Reinke et al 2021 [28]

Supplementary file2 (PDF 1471 KB) **Suppl Figure 2**: Hausdorff Distance (HD) and the 95% percentile (denoted as ${x}_{95}$) Hausdorff Distance 95% Percentile (HD95). The Euclidean distance between boundary pixels $a$ and $b$ is defined as $d(a,b)$. Only True Positive (TP) are considered. Figure courtesy of Reinke et al 2021 [28]

Supplementary file3 (PDF 232 KB) **Suppl Figure 3**: Probabilistic tractography of CN VII/VIII complex in healthy volunteers using conventional SS-EPI. Seed ROIs drawn on the hrT2 image and co-registered with the diffusion data. Representative image of tractography acquired with a probabilistic algorithm displayed on the subject’s co-registered hrT2 image (axial view through IAM). IAM and brainstem ROIs drawn on the hrT2 image and co-registered with the diffusion data. S: Subject, FOD-t: Fibre Orientation Distribution threshold. Note an aberrant tract in S1, left.

Supplementary file4 (PDF 732 KB) **Suppl Figure 4**: Colour tissue mapping and anatomical imaging of the facial nerve in patients with a vestibular schwannoma (P1 - P5). *Left*: High resolution T2-weighted MRI scan of vestibular schwannoma. Tractography results overlaid on P2 and P5 (tractography not successful in other patients). P1-4: axial hrT2 MRI, P5: coronal hrT2 MRI. *Right*: Corresponding CTM image delineating the facial nerve (arrow). Colour legend: *red*: WM; *green*: GM/tumour; *blue*: CSF/cystic fluid

## Abbreviations

ASSD: Average symmetric surface distance
CSD: Constrained spherical deconvolution
CTM: Colour tissue maps
dMRI: Diffusion magnetic resonance imaging
DWI: Diffusion-weighted imaging
EPI: Echo planar imaging
HD-95: 95% Haudsorff distance
IAM: Internal auditory meatus
rs-DWI: Readout-segmented diffusion-weighted imaging
VS: Vestibular schwannoma

## Footnotes

1 http://www.mrtrix.org

2 https://mrtrix.readthedocs.io/en/latest/constrained_spherical_deconvolution/multi_shell_multi_tissue_csd.html

## Declarations

### Guarantor

The scientific guarantor of this publication is Jonathan Shapey.

### Statistics and biometry

One of the authors has significant statistical expertise.

### Study subjects or cohorts overlap

Study subjects or cohorts have not been previously reported.

### Methodology

• Prospective

• Observational

• Performed at one institution
