# Supplementary material for: Diffusion MRI of the facial-vestibulocochlear nerve complex: a prospective clinical validation study
Source: Eur Radiol. 2023 Jun 17;33(11):8067–76. doi: 10.1007/s00330-023-09736-4 (PMC10598116; doi:10.1007/s00330-023-09736-4)
Supplement: Supplementary file 2 — Supplementary file2 (PDF 1471 KB) Suppl Figure 2: Hausdorff Distance (HD) and the 95% percentile (denoted as ${x}_{95}$x95) Hausdorff Distance 95% Percentile (HD95). The Euclidean distance between boundary pixels $a$a and $b$b is defined as $d(a,b)$d(a,b). Only True Positive (TP) are considered. Figure courtesy of Reinke et al 2021 [28] [file 330_2023_9736_MOESM2_ESM.pdf]

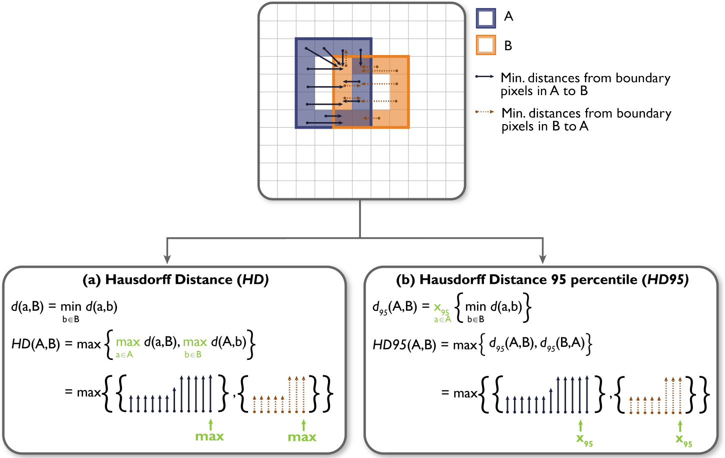
A
B
Min. distances from boundary pixels in A to B
Min. distances from boundary pixels in B to A
(a) Hausdorff Distance (HD)
d(a,B) = min d(a,b)
b∈B
HD(A,B) = max { max d(a,B), max d(A,b) }
a∈A b∈B
= max
max
max
(b) Hausdorff Distance 95 percentile (HD95)
d95(A,B) = x95 { min d(a,b) }
a∈A b∈B
HD95(A,B) = max { d95(A,B), d95(B,A) }
= max
x95
x95
