# Supplementary figures and images for: Diffusion MRI of the facial-vestibulocochlear nerve complex: a prospective clinical validation study
Source: Eur Radiol. 2023 Jun 17;33(11):8067–76. doi: 10.1007/s00330-023-09736-4 (PMC10598116; doi:10.1007/s00330-023-09736-4)

ss-EPI-1

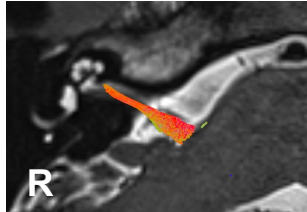

FOD-t: 0.1

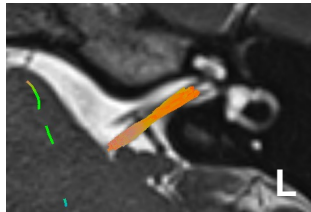

FOD-t: 0.03

ss-EPI-2

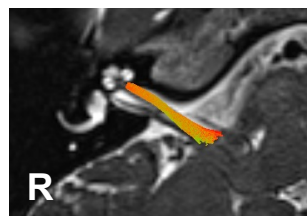

FOD-t: 0.

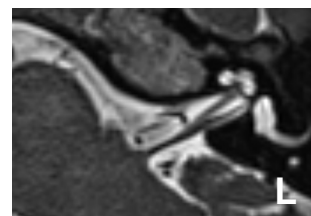

None

ss-EPI-3

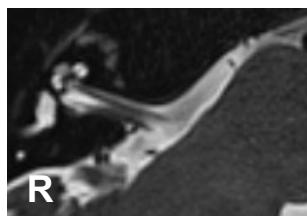

None

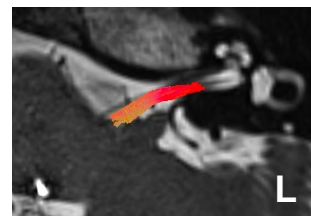

FOD-t: 0.1

ss-EPI-4

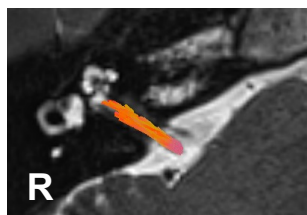

FOD-t: 0.6

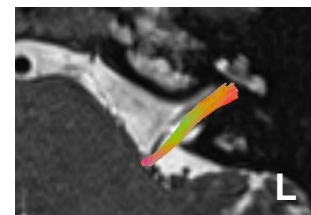

FOD-t: 0.8

ss-EPI-5

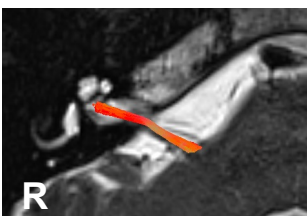

FOD-t: 0.8

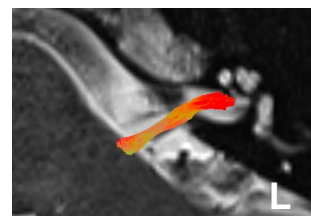

FOD-t: 0.6

Supplement: Supplementary file 3 — Supplementary file3 (PDF 232 KB) Suppl Figure 3: Probabilistic tractography of CN VII/VIII complex in healthy volunteers using conventional SS-EPI. Seed ROIs drawn on the hrT2 image and co-registered with the diffusion data. Representative image of tractography acquired with a probabilistic algorithm displayed on the subject’s co-registered hrT2 image (axial view through IAM). IAM and brainstem ROIs drawn on the hrT2 image and co-registered with the diffusion data. S: Subject, FOD-t: Fibre Orientation Distribution threshold. Note an aberrant tract in S1, left. [file 330_2023_9736_MOESM3_ESM.pdf]

P1

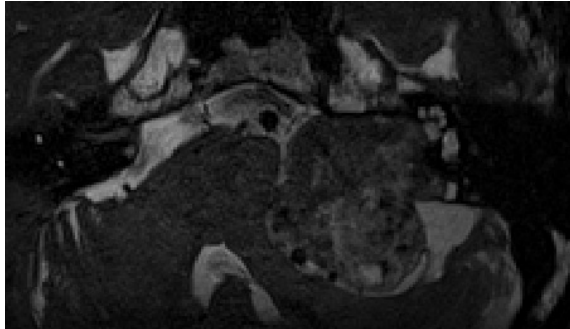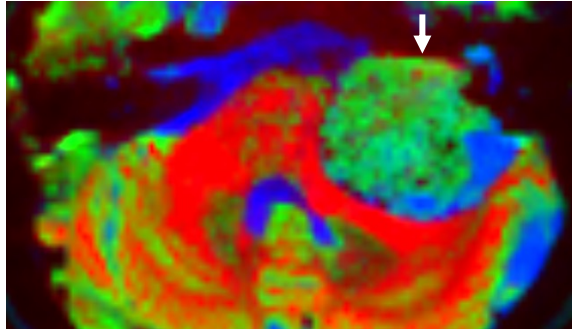

P2

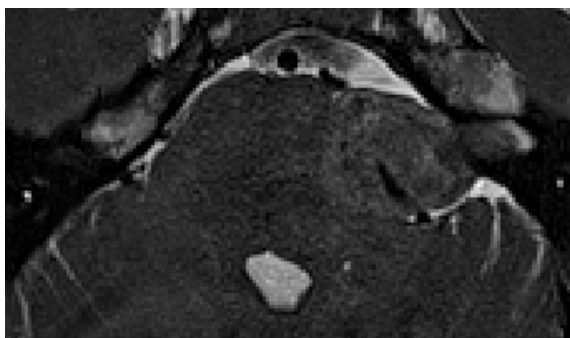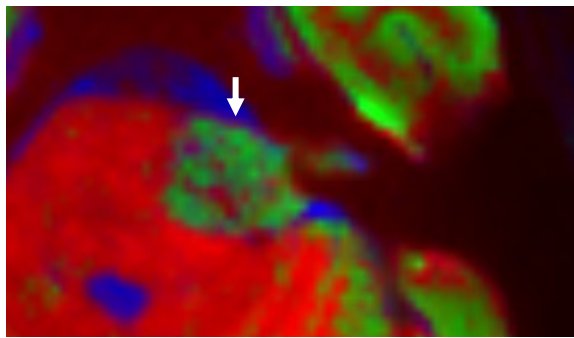

P3

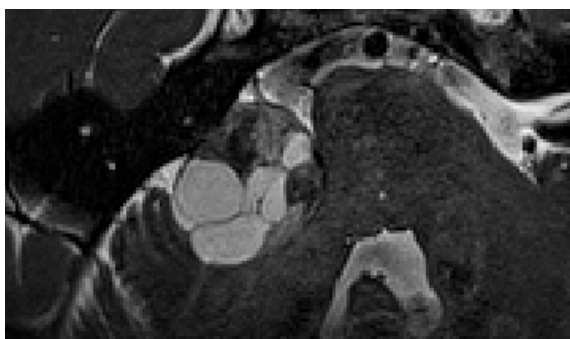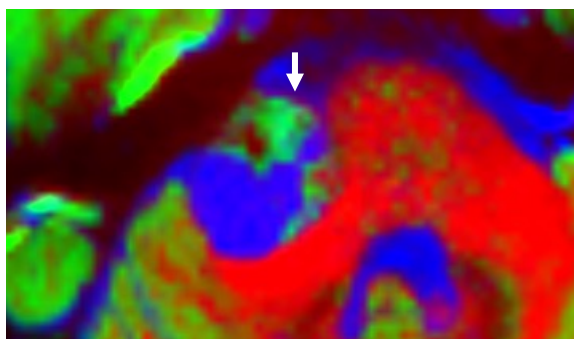

P4

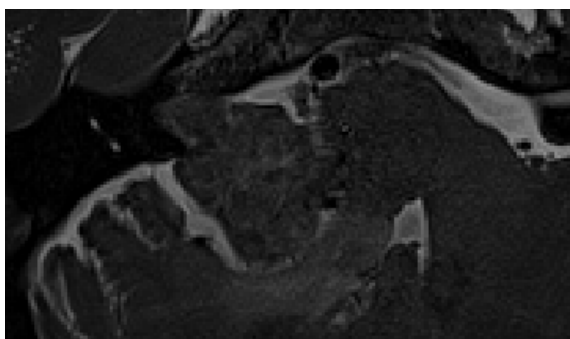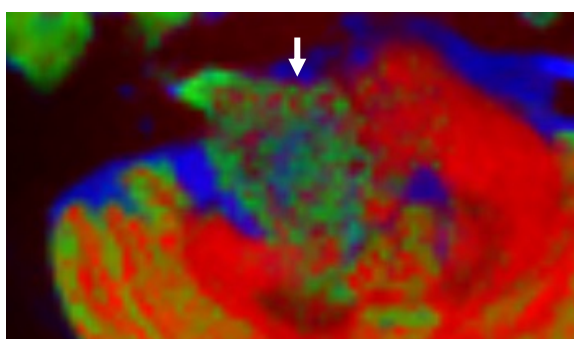

P5

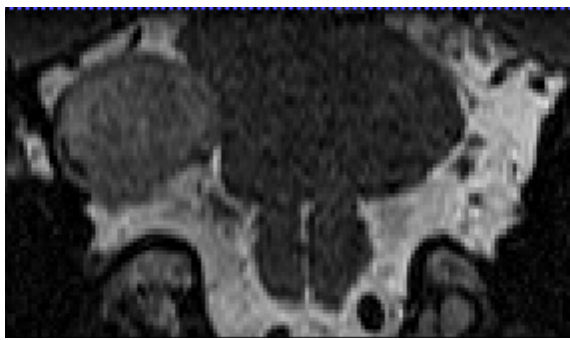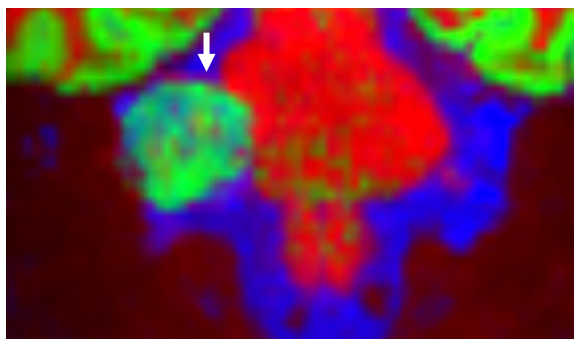

Supplement: Supplementary file 4 — Supplementary file4 (PDF 732 KB) Suppl Figure 4: Colour tissue mapping and anatomical imaging of the facial nerve in patients with a vestibular schwannoma (P1 - P5). Left: High resolution T2-weighted MRI scan of vestibular schwannoma. Tractography results overlaid on P2 and P5 (tractography not successful in other patients). P1-4: axial hrT2 MRI, P5: coronal hrT2 MRI. Right: Corresponding CTM image delineating the facial nerve (arrow). Colour legend: red: WM; green: GM/tumour; blue: CSF/cystic fluid [file 330_2023_9736_MOESM4_ESM.pdf]
