# Supplementary material for: Diffusion MRI of the facial-vestibulocochlear nerve complex: a prospective clinical validation study
Source: Eur Radiol. 2023 Jun 17;33(11):8067–76. doi: 10.1007/s00330-023-09736-4 (PMC10598116; doi:10.1007/s00330-023-09736-4)
Supplement: Supplementary file 1 — Supplementary file1 (PDF 1356 KB) Suppl Figure 1: Average Symmetric Surface Distance (ASSD) and Mean Average Surface Distance (MASD). The Euclidean distance between boundary pixels $a$a and $b$b is defined as $d(a,b)$d(a,b). Only True Positive (TP) are considered. Figure courtesy of Reinke et al 2021 [28] [file 330_2023_9736_MOESM1_ESM.pdf]

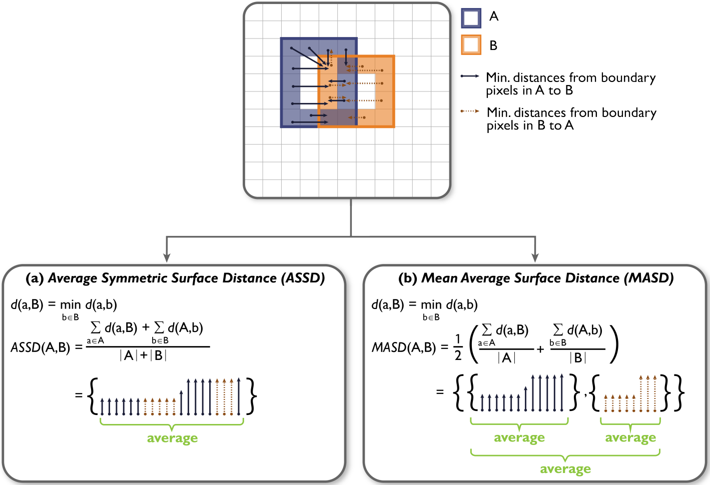

A
B
Min. distances from boundary pixels in A to B
Min. distances from boundary pixels in B to A
(a) Average Symmetric Surface Distance (ASSD)
$d(a,B) = \min_{b \in B} d(a,b)$
$ASSD(A,B) = \frac{\sum_{a \in A} d(a,B) + \sum_{b \in B} d(A,b)}{|A| + |B|}$
average
(b) Mean Average Surface Distance (MASD)
$d(a,B) = \min_{b \in B} d(a,b)$
$MASD(A,B) = \frac{1}{2}\left(\frac{\sum_{a \in A} d(a,B)}{|A|} + \frac{\sum_{b \in B} d(A,b)}{|B|}\right)$
average
average
average
